# Cardiovascular magnetic resonance reference ranges for the heart and aorta in Chinese at 3T

**DOI:** 10.1186/s12968-016-0236-3

**Published:** 2016-04-12

**Authors:** Thu-Thao Le, Ru San Tan, Michelle De Deyn, Elizabeth Pee Chong Goh, Yiying Han, Bao Ru Leong, Stuart Alexander Cook, Calvin Woon-Loong Chin

**Affiliations:** National Heart Centre Singapore, 5 Hospital Drive, Singapore, 169609 Singapore; Trinity College Dublin, School of Medicine, Dublin, Republic of Ireland

**Keywords:** Cardiovascular magnetic resonance, Normal reference ranges, Ventricular volumes, Ventricular function

## Abstract

**Background:**

Cardiovascular magnetic resonance (CMR) reference ranges have not been well established in Chinese. Here we determined normal cardiac and aortic reference ranges in healthy Singaporean Chinese and investigated how these data might affect clinical interpretation of CMR scans.

**Methods:**

In 180 healthy Singaporean Chinese (20 to 69 years old; males, *n* = 91), comprehensive cardiac assessment was performed using the steady state free precision technique (3T Ingenia, Philips) and images were analysed by two independent observers (CMR42, Circle Cardiovascular Imaging). Measurements were internally validated using standardized approaches: left ventricular mass (LVM) was measured in diastole and systole (with and without papillary muscles) and stroke volumes were compared in both ventricles. All reference ranges were stratified by sex and age; and “indeterminate/borderline” regions were defined statistically at the limits of the normal reference ranges. Results were compared with clinical measurements reported in the same individuals.

**Results:**

LVM was equivalent in both phases (mean difference 3.0 ± 2.5 g; *P* = 0.22) and stroke volumes were not significantly different in the left and right ventricles (*P* = 0.91). Compared to females, males had larger left and right ventricular volumes (*P* < 0.001 for all). Indexed LVM was significantly higher in males compared to females (50 ± 7 versus 38 ± 5 g/m^2^, respectively; *P* < 0.001). Overall, papillary muscles accounted for only ~2 % of the total LVM. Indexed atrial sizes and aortic root dimensions were similar between males and females (*P* > 0.05 for all measures). In both sexes, age correlated negatively with left and right ventricular volumes; and positively with aortic sinus and sinotubular junction diameters (*P* < 0.0001 for all). There was excellent agreement in indexed stroke volumes in the left and right ventricles (0.1±5.7mL/m^2^, 0.7±6.2 mL/m^2^, respectively), LVM (0.6±6.4g/m^2^), atrial sizes and aortic root dimensions between values reported in clinical reports and our measured reference ranges.

**Conclusions:**

Comprehensive sex and age-corrected CMR reference ranges at 3T have been established in Singaporean Chinese. This is an important step for clinical practice and research studies of the heart and aorta in Asia.

**Electronic supplementary material:**

The online version of this article (doi:10.1186/s12968-016-0236-3) contains supplementary material, which is available to authorized users.

## Background

An accurate assessment of cardiac volumes, function and mass is crucial in the diagnosis, management and prognosis of patients with cardiovascular diseases [1, 2]. Although echocardiography is widely used and well studied, it relies heavily upon suitable echocardiographic windows, experience of the operator and a series of geometrical and mathematical assumptions in estimating ventricular volumes and mass [3]. On the other hand, assessment by cardiovascular magnetic resonance (CMR) offers highly accurate and reproducible measures of the left and right ventricles. The excellent scan-rescan reproducibility also translates to a significant reduction in sample sizes required for clinical studies [4]. These advantages have propelled CMR as the standard reference for assessing cardiac morphology and function [5, 6].

Unfortunately, a vast majority of CMR reference ranges have been established in healthy individuals from the West [7–10] and they may not be representative of the diverse world population to which these values are applied in. Indeed, cardiac dimensions and function have not been well defined in Asia. In particular, there are no studies examining CMR reference ranges for atrial sizes and aortic root dimensions in Asia despite their important prognostic implications [11–13].

As the indications of CMR continues to expand in Asia [2], there remains an urgent need to establish reference ranges in order to confidently differentiate abnormal from normal phenotypes. In this study, we set out to define comprehensive age and sex specific reference ranges for left and right ventricular and atrial dimensions, left ventricular mass (LVM) and aortic root dimensions in healthy Singaporean Chinese. In the same cohort of healthy individuals, we compared our reference ranges with clinically derived measurements and examined the potential impact of adopting our newly derived ranges in the local setting.

## Methods

### Patient population

Singaporean Chinese (20 to 69 years old) without symptoms, clinical or family history of cardiovascular or cerebrovascular disease were prospectively recruited from the community by advertisement in local media. Volunteers did not have hypertension, hyperlipidemia, or diabetes mellitus. Any volunteer with valvular heart disease or wall motion abnormalities detected on CMR was excluded from the analysis. In order to ensure an adequate distribution of patients across the age range, 15 to 20 individuals were systematically recruited in each age decile in either sex.

The study was conducted in accordance with the Declaration of Helsinki and approved by the Singhealth Centralised Institutional Review Board. Written informed consent was obtained from all patients.

### Cardiovascular magnetic resonance

CMR was performed in all patients on a 3T scanner (Ingenia, Philips Healthcare, Best, the Netherlands). Balanced steady state free precision (SSFP) cines were acquired in the vertical and horizontal long axis planes, right ventricular (RV) long axis view that is aligned with the tricuspid inflow and RV outflow tract view, as well as, the sagittal left ventricular outflow tract view (TR 2.8 to 2.9 ms; TE 1.4 to 1.5 ms; turbo factor = 10; acquired voxel size 1.88 × 1.90 × 8.00 mm^3^, flip angle 45°; 40 phases per cardiac cycle). Subsequent short axis cines extending from the atrioventricular ring to the apex were obtained to cover the entire left and right ventricles (8 mm parallel slices with 2 mm gap; acquired voxel size 1.89 × 1.83 × 8.00 mm^3^; 30 phases per cardiac cycle).

### Image analysis

**Fig. 1:**
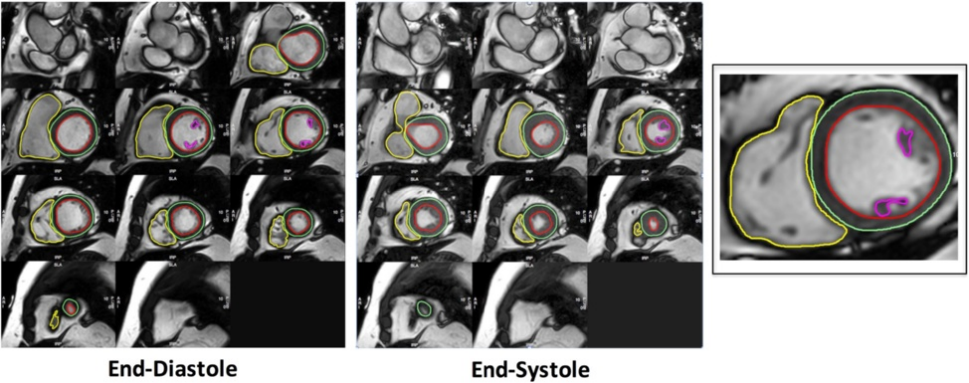
Contouring of Left and Right Ventricles in Diastole and Systole. Endo- and epicardial borders of the left ventricle were contoured in end-diastole and end-systole. In the left ventricle, papillary muscles were included in myocardial mass assessment and excluded from volumes estimation. Left ventricular mass was corroborated in end-diastole and end-systole. In the right ventricle, trabeculations and papillary muscles were included as part of the ventricular cavity so as to improve reproducibility

**Fig. 2:**
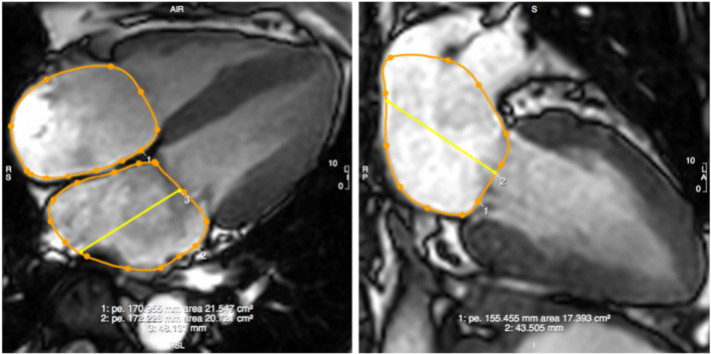
Contouring of Left and Right Atrial Dimensions. Maximal left atrial volume is estimated using the biplane area-length method (LAV = [8 x (2-chamber area) x (4-chamber area)]/3πL, where L is the shorter of the two left atrial length in the two views). Measurements were taken in the four- and two-chamber views, at the end of ventricular systole (a frame before opening of the mitral valve). Pulmonary veins and atrial appendage were excluded. Left atrial length was measured from the mid-point of the mitral annulus to the superior aspect of the left atrium. Right atrial area was determined in the four-chamber view

**Fig. 3:**
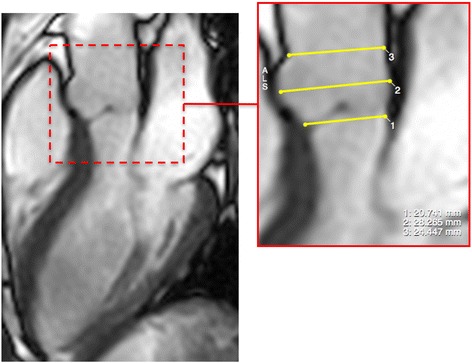
Contouring of Aortic Annulus and Root Dimensions. Aortic annulus, sinus and sinotubular junction dimensions were measured at the end of ventricular diastole (insert) in the sagittal left ventricular outflow tract view

The analysis of cardiac volumes (end-diastolic EDV; end-systolic ESV), function and mass was performed in our research image analysis laboratory using standardized protocols (Figs. 1, 2 and 3) and a dedicated software (CMR42, Circle Cardiovascular Imaging, Calgary, Alberta, Canada) [14–16]. For the analysis of left ventricular (LV) volumes, the LV outflow tract (extending up to the aortic cusps) was included as part of the blood volume. The identification of LV basal slice was facilitated with the horizontal long axis and the sagittal LV outflow tract views. Papillary muscles were excluded in the estimation of left ventricular volumes. LVM was estimated at end-diastole and corroborated at end-systole: (total epicardial volume - total endocardial volume) x 1.05 g/ml; and mass was reported with and without the inclusion of papillary muscles (Fig. 1). In the RV, volumes below the pulmonary valve were included. The identification of the RV basal slice was corroborated with the horizontal long axis and RV long axis views. Trabeculations and papillary muscles of the RV were included as part of the ventricular cavity and a smooth endocardial border is drawn to improve reproducibility [14] (Fig. 1).

The estimation of maximal left atrial (LA) volumes was based on the biplane area-length method. In both the vertical and horizontal long axis views at the end of ventricular systole (at the frame before mitral valve opening), LA length was measured from the midpoint of the mitral annulus plane to the posterior aspect of the left atrium. The LA area was carefully contoured to exclude the pulmonary veins and left atrial appendage [12] (Fig. 2). Right atrial (RA) area was measured at the end of ventricular systole (same frame used to assess LA dimensions) in the horizontal long axis view (Fig. 2).

The measurement of aortic root dimensions was performed at the end of ventricular diastole in the sagittal left ventricular outflow tract view. Measurements of the aortic root were made at three levels: the level of aortic annulus, across the sinus and at the sinotubular junction [15] (Fig. 3).

Height and weight were measured in all patients and body surface area (BSA) was calculated using the DuBois formula [16]. Absolute and BSA-indexed volumes, LVM and cardiac dimensions would be reported in the study. Two experienced operators (LTT and CWLC) analyzed all the scans in this study.

### Statistical analysis

The distribution of all continuous variables was assessed for normality using the Shapiro-Wilk test and presented as either mean ± standard deviation or median [interquartile range], as appropriate. Simple linear regression was used to model the association between cardiac measurements and age. The reference range is defined as the 95 % prediction interval: 95 % prediction interval = mean ± t_0.975, n-1_ (√(n + 1)/n)(standard deviation). To account for the effects of sample size on the reference range, 95 % confidence intervals of the (upper and lower) reference limits were also estimated [17]. Values within these confidence intervals were considered “indeterminate or borderline”. All statistical analyses were performed using GraphPad Prism 6 (GraphPad Software, Inc., San Diego, CA). A 2-sided *P* < 0.05 was considered statistically significant.

## Results

CMR scans of 180 individuals (males, *n* = 91; 45 ±13 years old) were analyzed and the cardiac measurements (absolute and BSA-indexed values, where applicable) of the cohort were summarized in Table 1.

**Table 1:** Clinical characteristics and CMR measurements

	All patients
	(*n* = 180)
Clinical Characteristics	
Age, years	45 ± 13
Males, n (%)	91 (51)
Systolic blood pressure, mmHg	130 ± 16
Diastolic blood pressure, mmHg	79 ± 11
Heart rate, beats per min	76 ± 12
Weight, kg	64 ± 13
Height, m	1.65 ± 0.09
Body surface area, m^2^	1.70 ± 0.20
Body mass index, kg/m^2^	23.3 ± 3.5
Left Ventricle Measurements	
Absolute Values	
LV mass (no papillary muscles), g	76 ± 22
LV mass (with papillary muscles), g	78 ± 22
LV end-diastolic volume, mL	128 ± 28
LV end-systolic volume, mL	51 ± 14
LV stroke volume, mL	77 ± 16
LV ejection fraction, %	60 ± 5
Values Indexed to body surface area	
Indexed LV mass (no papillary muscles), g/m^2^	44 ± 9
Indexed LV mass (with papillary muscles), g/m^2^	45 ± 9
Indexed LV end-diastolic volume, mL/m^2^	75 ± 12
Indexed LV end-systolic volume, mL/m^2^	30 ± 7
Indexed LV stroke volume, mL/m^2^	45 ± 7
Right Ventricle Measurements	
Absolute Values	
RV end-diastolic volume, mL	143 ± 35
RV end-systolic volume, mL	67 ± 22
RV stroke volume, mL	77 ± 16
RV ejection fraction, %	54 ± 7
Values Indexed to body surface area	
Indexed RV end-diastolic volume, mL/m^2^	84 ± 15
Indexed RV end-systolic volume, mL/m^2^	39 ± 11
Indexed RV stroke volume, mL/m^2^	45 ± 7
Left and Right Atrial Measurements	
Absolute Values	
LA area (4 chamber), cm^*2*^	22 ± 4
LA area (2 chamber), cm^2^	19 ± 3
LA volume, mL	85 ± 20
RA area (4 chamber), cm^2^	23 ± 4
Values Indexed to body surface area	
Indexed LA area (4 chamber), cm^2^/m^2^	13 ± 2
Indexed LA area (2 chamber), cm^2^/m^2^	11 ± 2
Indexed LA volume, mL/m^2^	50 ± 10
Indexed RA area (4 chamber), cm^2^/m^2^	12 ± 2
Aortic Annulus and Root Measurements	
Absolute Values	
Annulus diameter, mm	21 ± 2
Sinus diameter, mm	29 ± 4
Sinotubular junction, mm	23 ± 4
Values indexed to body surface area	
Indexed annulus diameter, mm/m^2^	12 ± 1
Indexed sinus diameter, mm/m^2^	17 ± 2
Indexed sinotubular junction, mm/m^2^	14 ± 2

(Abbreviations: *LV* left ventricle, *RV* right ventricle, *LA* left atrium, *RA* right atrium)

### Left ventricular dimensions and function according to sex and age

Absolute and indexed LV EDV and ESV were larger in males compared to females (*P* < 0.0001 for all). Absolute and indexed LVM were significantly increased in males compared to females (*P* < 0.0001 for both). Papillary muscles contributed little to overall LVM (1.7 ± 0.7 g; accounting for 2.2 % of the total left ventricular mass). Similar LVM measured in diastolic and systolic phases (mean difference 3.0 ± 2.5 g; correlation *r* = 0.99; *P* < 0.0001) provided internal validation of the accuracy of our methods.

**Fig. 4:**
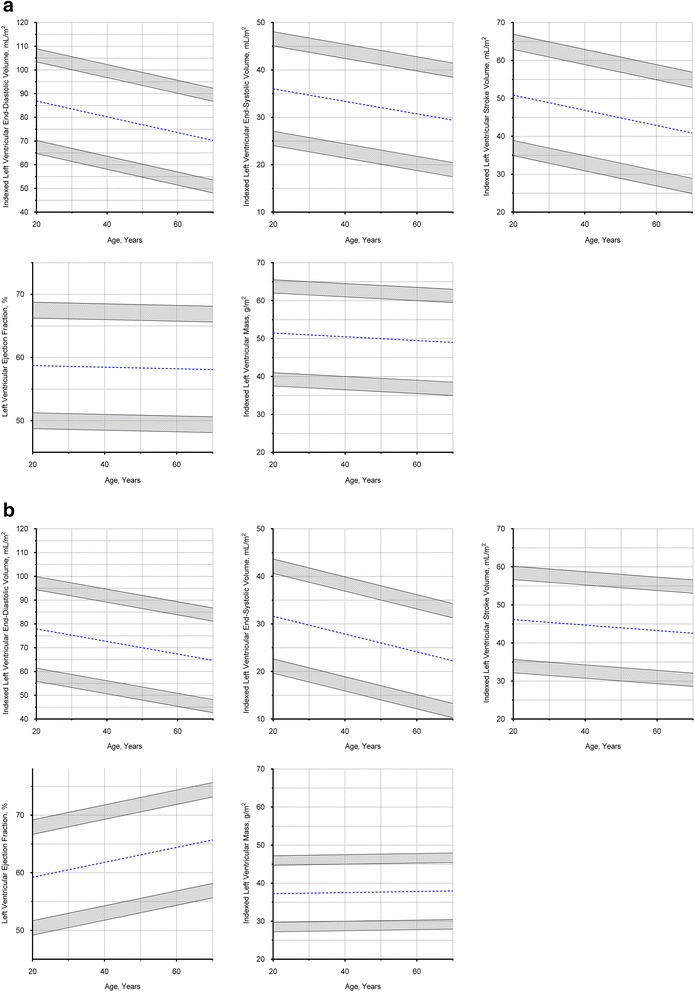
Left ventricular Dimensions in Males and Females. **a** Males, values in the shaded regions are indeterminate abnormal or borderline normal. **b** Females, values in the shaded regions are indeterminate abnormal or borderline normal

LV volumes correlated negatively with age in both males (EDV: *r* = -0.45; *P* < 0.001; ESV: *r* = -0.36; *P* < 0.001) and females (EDV: *r* = -0.33; *P* < 0.001; ESV: *r* = -0.44; *P* < 0.001), with similar correlations after normalizing to BSA (*P* < 0.001 for all). Whilst LV ejection fraction correlated positively with age in females (*r* = 0.38; *P* < 0.001), there was no correlation with age in males (*r* = -0.03; *P* = 0.75). No correlation between LVM and age was observed in both males (absolute mass: *r* = -0.17; *P* = 0.10; indexed mass: *r* = -0.09; *P* = 0.39) and females (absolute mass: *r* = -0.05; *P* = 0.67; indexed mass: *r* = 0.04; *P* = 0.72). Age-specific reference ranges for ventricular measurements in both males and females were established (Fig. 4; Tables 2 and 3).

**Table 2:**
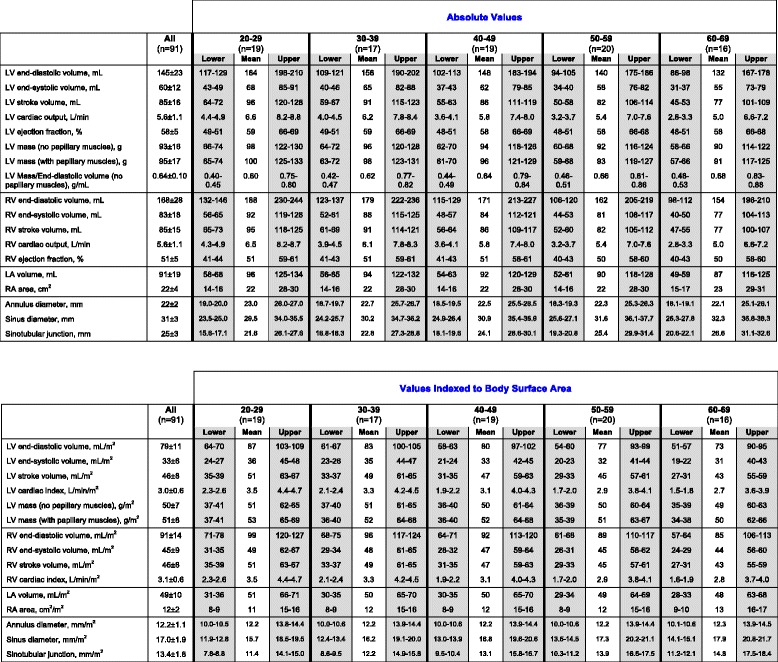
Absolute and indexed cardiac dimensions in males

Values in the upper and lower confidence interval of reference limits (grey columns) are “indeterminate abnormal or borderline normal”

**Table 3:**
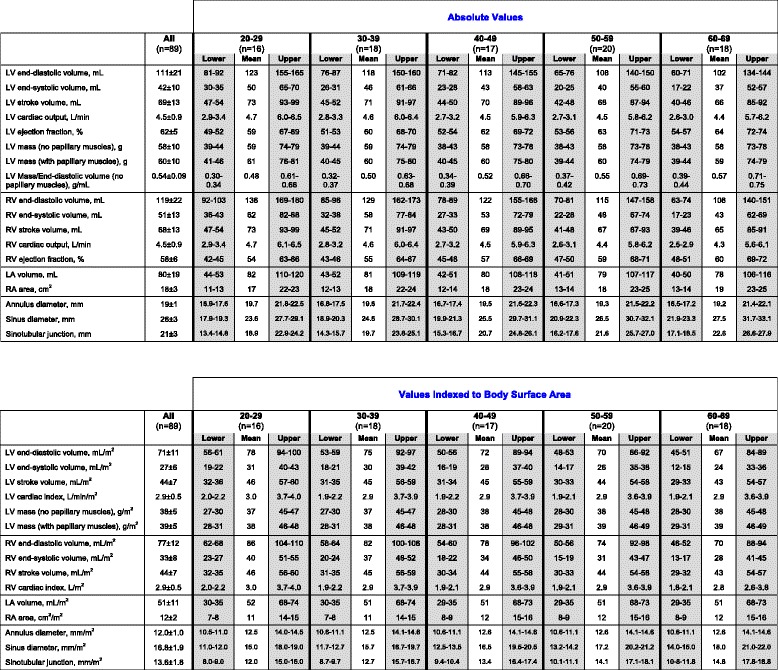
Absolute and indexed cardiac dimensions in females

Values in the upper and lower confidence interval of reference limits (grey columns) are “indeterminate abnormal or borderline normal”

### Right ventricular dimensions and function according to sex and age

**Fig. 5:**
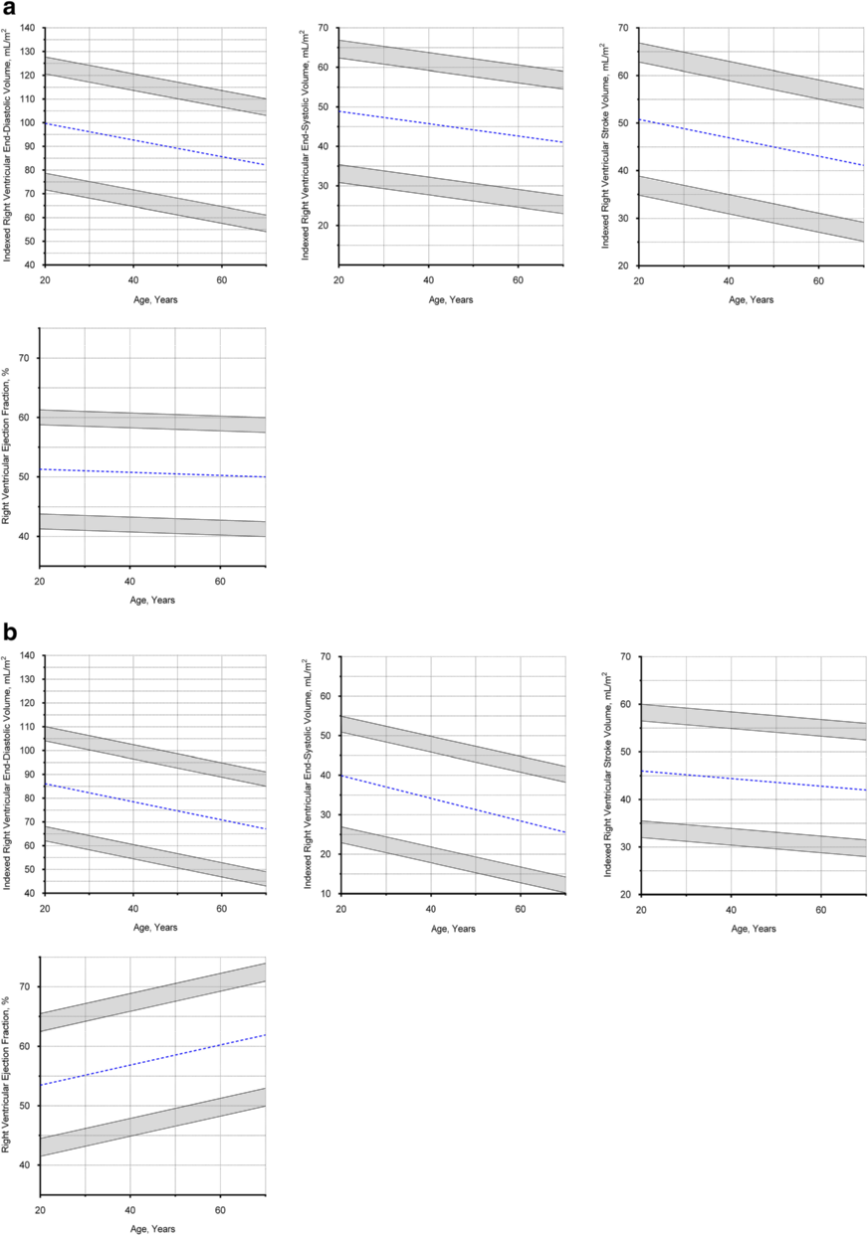
Right ventricular Dimensions in Males and Females. **a** Males, values in the shaded regions are indeterminate abnormal or borderline normal. **b** Females, values in the shaded regions are indeterminate abnormal or borderline normal

Similar findings with sex and age were observed in the RV (Fig. 5; Tables 2 and 3). Absolute RV EDV and ESV were significantly larger in males compared to females (EDV: 168 ± 28 versus 119 ± 22 mL, respectively; *P* < 0.001; ESV: 83 ± 18 versus 51 ± 13 mL, respectively; *P* < 0.001), and remained significantly different after normalizing to BSA (*P* < 0.001 for all). Similar stroke volumes measured in the right and left ventricles (77 ± 16 versus 77 ± 16, respectively; *P* = 0.99) underscored the internal validity of our methods.

RV volumes correlated negatively with age in both males (EDV: *r* = -0.41; *P* < 0.001; ESV: *r* = -0.30; *P* < 0.01) and females (EDV: *r* = -0.44; *P* < 0.001; ESV: *r* = -0.50; *P* < 0.001), with similar correlations after normalizing to BSA (*P* < 0.05 for all). Similar to the LV, RV ejection fraction correlated modestly with age in females (*r* = 0.36; *P* < 0.001) but not in males (*r* = -0.07; *P* = 0.53) (Fig. 5).

### Left and right atrial dimensions according to sex and age

**Fig. 6:**
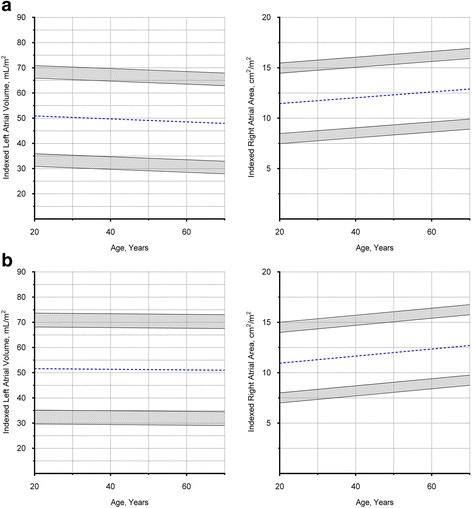
Atrial Dimensions in Males and Females. **a** Males, values in the shaded regions are indeterminate abnormal or borderline normal. **b** Females, values in the shaded regions are indeterminate abnormal or borderline normal

Males had larger absolute LA volumes and RA areas compared to females (LA volume: 91 ± 19 versus 80 ± 19 mL, respectively; *P* < 0.001; RA area: 22 ± 4 versus 18 ± 3 cm^2^, respectively; *P* < 0.001), but not when normalized to BSA (Indexed LA volume: 49 ± 10 versus 51 ± 11 mL/m^2^, respectively; *P* = 0.24; Indexed RA area: 12 ± 2 versus 12 ± 2 cm^2^, respectively; *P* = 0.26). There was no correlation between indexed LA volumes and age in either males (*r* = -0.08; *P* = 0.45) or females (*r* = -0.02; *P* = 0.89). Conversely, indexed RA area correlated weakly with age in either sex (females: *r* = 0.27; *P* = 0.01; males: *r* = 0.18; *P* = 0.09) (Fig. 6).

### Aortic root dimensions according to sex and age

**Fig. 7:**
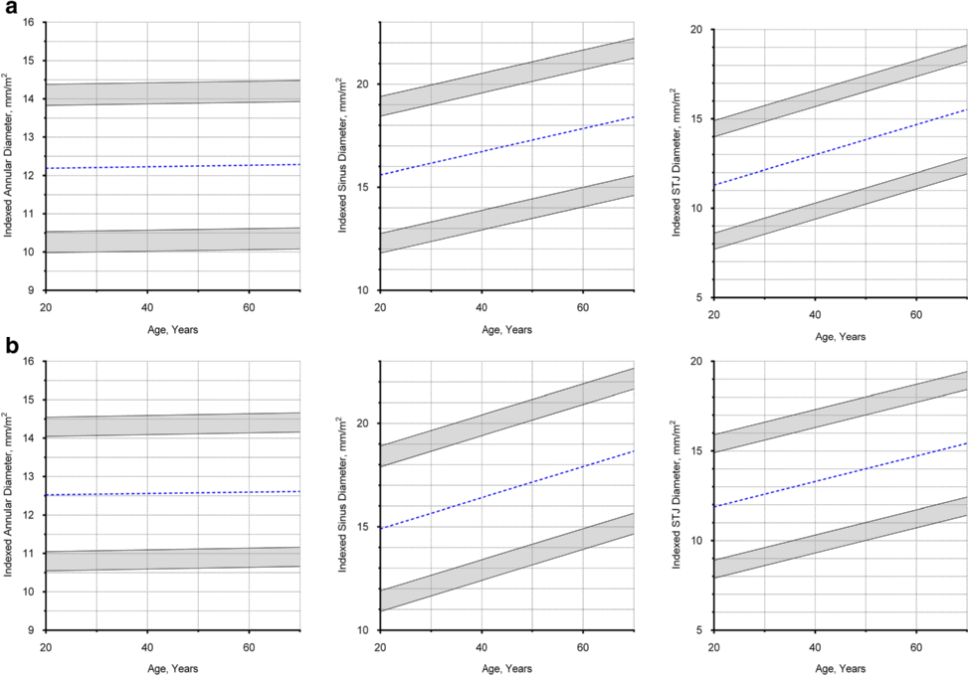
Aortic Root Dimensions in Males and Females. **a** Males, values in the shaded regions are indeterminate abnormal or borderline normal. **b** Females, values in the shaded regions are indeterminate abnormal or borderline normal

Males have larger absolute annular and aortic root dimensions compared to females (*P* < 0.001 for all), but not when normalized to BSA (*P* > 0.10 for all) (Tables 2 and 3). There was a moderate to strong correlation between indexed aortic root dimensions and age in males (sinus: *r* = 0.39; *P* < 0.0001; STJ: *r* = 0.64; *P* < 0.0001) and females (sinus: *r* = 0.53; *P* < 0.0001; STJ: *r* = 0.54; *P* < 0.0001). In contrast, there was no correlation between annular diameter (absolute or indexed values) and age in either sex (*r* = 0.02; *P* = 0.83 for both) (Fig. 7).

### Inter-observer reproducibility of cardiac measurements

The reproducibility of our analysis techniques was tested in 10 individuals (males, *n* = 5). We were able to achieve excellent inter-operator reproducibility in the LV (EDV: 4.0 ± 4.6 mL; ESV: 2.6 ± 2.9 mL; LVM: 1.9 ± 1.7 g), RV (EDV: 1.1 ± 3.9 mL; ESV: 2.1.0 ± 5.0 mL), atrial (LA volume: 2.7 ± 4.6 mL; RA area: 0.4 ± 0.7 cm^2^) and aortic root (aortic annulus: 0.4 ± 1.0 mm; sinus: 0.1 ± 0.7 mm; STJ: 0.4 ± 0.7 mm) measurements.

### Comparison with real-world data

At our center, all clinically acquired CMR images have to date been analysed by trained cardiology Fellows and radiographers using dedicated software (IntelliSpace Portal (ISP) Version 6.0.3, Philips Healthcare, Best, the Netherlands) and clinical reports archived on clinical systems from which quantitative analyses can be extracted. Using ISP, papillary muscles are excluded from LVM and included in LV volumes. In the same cohort of 180 healthy individuals reported above, cardiac dimensions measured clinically/in the real world (involving approximately 6 Fellows and radiographers) were retrieved from our clinical database and compared with our reference ranges.

**Fig. 8:**
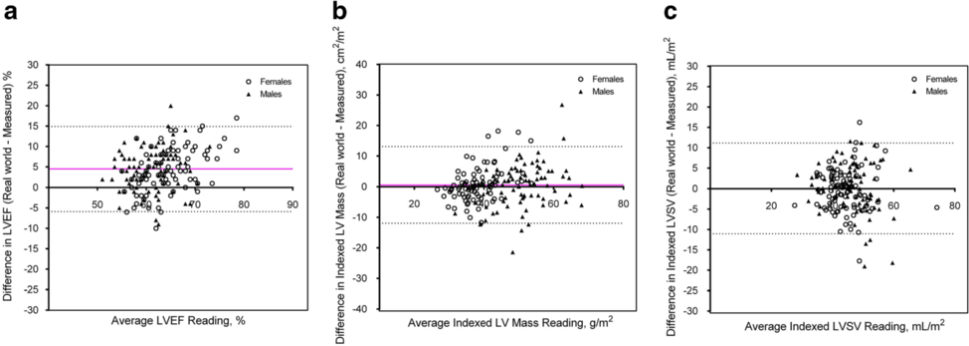
Comparison of Real World Values and Reference Range Measurements Established in the Study. **a** Left Ventricular Ejection Fraction; **b** Indexed Left Ventricular Mass and (**c**) Indexed Stroke Volume

Compared to values in the clinical reports, our reference range ventricular volumes were larger (see Additional file 1). Despite this difference, there was excellent agreement in indexed stroke volumes (LV: 0.1 ± 5.7 mL/m^2^; RV: -0.7 ± 6.2 mL/m^2^; Fig. 8). Although there was a systematic overestimation of clinically reported LV ejection fraction (5 ± 5 %), none was below the lower limits defined in our study. There was also excellent agreement in indexed LVM (0.6 ± 6.4 g/m^2^), atrial areas (LA: 1.7 ± 1.2 cm^2^/m^2^; RA: 1.7 ± 1.3 cm^2^/m^2^) and aortic root dimensions (sinus: -0.3 ± 1.2 mm/m^2^) (Fig. 8; see Additional file 1).

## Discussion

Here we systematically assessed dimensions of the left and right ventricles, left and right atria and aortic root in a cohort of healthy volunteers, the first and the largest Asian study of its kind at 3T. We observed sex- and age-related differences in cardiac volumes and LVM, as expected. We assessed LVM with/without papillary muscles and for the first time, demonstrated a small effect of papillary muscles on the total left ventricular mass in healthy individuals. Finally, we compared our reference ranges with clinically reported/real world measurements. Real world data demonstrated good overall agreement with our reference ranges.

In the study, we used multiple robust approaches to internally validate cardiac measurements and had achieved excellent inter-operator reproducibility. We assessed and demonstrated similar left and right ventricular stroke volumes in a single cohort of healthy individuals (*P* = 0.91). Indeed, in the absence of intra- or extracardiac shunts, left and right stroke volumes should be nearly equal [14]. Moreover, we have observed excellent correlation between volumetric stroke volumes in the LV with those obtained from phase contrast imaging (*r* = 0.84; *P* < 0.0001; see Additional file 2). Finally, we compared LVM in end-diastole and end-systole (78 ± 22 versus 80 ± 23 g, respectively; *P* = 0.22), which is expected to be similar because the effects of blood content on mass is small [18].

Clinicians rely on reference ranges to confidently differentiate normal and abnormal cardiac dimensions and function. By convention, reference ranges for a particular measurement are defined as the interval that contains 95 % of the values in a specific population assessed. Therefore, the precision is highly dependent on the study sample size, particularly at the boundaries of the reference limits [17]. Taking into account these uncertainties, we establish confidence intervals of the upper and lower reference limits. Values within these regions would be “indeterminate abnormal” or “borderline normal” that represents a grey-zone for clinical interpretation [19].

Compared to Caucasians, our Singaporean Chinese population had smaller cardiac volumes (LV EDV: 128 ± 28 versus 146 mL; RV EDV: 143 ± 35 versus 162 mL) and lower LVM (76 ± 22 versus 116 g); this difference remained after normalizing measurements to body surface area [20]. Therefore, there are important clinical implications if reference ranges established in Caucasian populations were to be adopted locally. Similar to reference ranges established in Caucasian populations, we observed negative correlations between ventricular volumes and age; and no associations between LVM and age [7, 20, 21]. Whilst studies in Caucasian populations showed positive correlation only between RV ejection fraction (but not LV ejection fraction) and age in both sexes [7, 21], we demonstrated a correlation between ejection fractions (LV and RV) and age only in females. Although indexed LA volumes in Singaporeans were higher than values reported in a Caucasian population (50 ± 10 versus 40 mL/m^2^), the findings have to be interpreted with caution because of differences in analysis methods [22]. Indexed RA area and aortic root dimensions in Singaporean Chinese were similar compared to Caucasians [15, 20]. Of interest, we observe a weak correlation between age and RA area, but not with LA size. Unlike the LA that remained relatively stable [23], RA enlargement likely occurs in response to RV diastolic dysfunction and increased RA pressure associated with increasing age [24]. Nevertheless, this weak association is unlikely to be of any clinical significance (difference of 1-2 cm^2^/m^2^ in RA size between the first (20-29) and last (60-69) age decile).

To the best of our knowledge, this is the first study that defines LVM reference ranges, with and without papillary muscles. Contrary to a previous study performed using fast gradient echo (FGE) sequence [25], we have demonstrated papillary muscles accounted for only 2 % of the total LVM (less than 2 g) and this is unlikely to be of any important clinical significance in healthy individuals. Our novel finding reflects the greater spatial resolution with newer CMR techniques. Indeed, SSFP is the current standard to assess myocardial function and mass because of improved blood-myocardium contrast over FGE. Moreover compared to 1.5 T, SSFP at 3 T has further increased signal-to-noise ratio for myocardium and blood, as well as, myocardium-to-blood contrast-to-noise ratio [26].

Unlike a recent Asian study at 1.5 T, we have reported the association between cardiac dimensions and age stratified by sex [27]. This has important clinical implications because there are sex-related differences in the association between some cardiac dimensions and age, as demonstrated in our study. We also observed differences in cardiac volumes and LV mass between our study and the previous reported Asian study that may reflect variabilities in contouring techniques. Nevertheless, the inter-study mean difference in cardiac volumes and LV mass between the two Asian studies (1 to 15 %) was less than that reported in other Caucasian studies (5 to 32 %) [7, 10, 21, 28].

The comparison between local reference ranges and real world measurements have not previously been reported. In many large CMR centers, image analyses are performed by Fellows and/or radiographers. In addition to variation in contouring techniques by different operators, current software packages differ with regards to the inclusion/exclusion of papillary muscles when estimating LV mass and volumes. These factors may limit the applicability of our reference ranges that were established under the most ideal conditions: robust cross-validation techniques and excellent inter-operator reproducibility. It was perhaps not unexpected to find differences between our reference ranges and the clinically reported values. However, we observed excellent agreement in terms of ventricular stroke volumes, LV mass, atrial and aortic root dimensions. Whilst LV ejection fraction assessed in the real world were systematically higher compared to those measured here, no clinically reported values fell below the lower limits established in our study. These findings support the overall adoption of our reference ranges locally, across analysis platforms and by all operators.

### Study limitations

The aortic root dimensions were measured only in the sagittal LV outflow tract view, not in the coronal LV outflow tract view or axial aortic sinus plane (these additional views were not obtained). However, the measurements in the sagittal LV outflow tract view remain the most relevant in current practice [3, 29]. We have not assessed RV mass because of the thin myocardium (2.7 ± 0.5 mm assessed in 10 healthy volunteers) and limited spatial resolution (acquired voxel size of approximately 2 mm). Of note, the challenge of assessing RV mass was also highlighted by other investigators [28]. For practical reasons, we do not routinely acquire the entire short-axis slices of the left and right atria to measure LA and RA volumes. This is to minimize scanning time and discomfort of the patients. However, the biplane area-length method of estimating LA volume was well validated to have important prognostic value [12] and RA area demonstrated excellent correlation with RA volume [30].

## Conclusions

In Singaporean Chinese, we have established comprehensive CMR reference ranges for the heart and aortic root that will have important clinical and research applications in Asia.

## Additional files

Additional file 1: Comparison of real world data and reference ranges established in the study. (PDF 30 kb) Additional file 2: Correlation between volumetric and phase contrast stroke volumes. (PDF 419 kb)

## Abbreviations

BSA: Body surface area
CMR: Cardiovascular magnetic resonance
EDV: End diastolic volume
ESV: End systolic volume
FGE: Fast gradient echo
LA: Left atrial
LV: Left ventricular/left ventricle
LVM: Left ventricular mass
RA: Right atrial
RV: Right ventricular/right ventricle
SSFP: Steady state free precision
STJ: Sino-tubular junction
