# Supplementary material for: Cardiovascular magnetic resonance reference ranges for the heart and aorta in Chinese at 3T
Source: J Cardiovasc Magn Reson. 2016 Apr 12;18:21. doi: 10.1186/s12968-016-0236-3 (PMC4830061; doi:10.1186/s12968-016-0236-3)
Supplement: Additional file 1: — Comparison of real world data and reference ranges established in the study. (PDF 30 kb) [file 12968_2016_236_MOESM1_ESM.pdf]

Real-world CMR Measurements

	(A) Measured Data	(B) Real-world Data	(B-A) / Mean (A+B)
**CMR LV Measurements**			
Absolute values			
LV mass (exclude papillary muscles), g	76±22	76±24	1±11
LV end-diastolic volume, mL	128±28	120±26	-9±10
LV end-systolic volume, mL	51±14	43±14	-9±7
LV stroke volume, mL	77±17	77±16	0.1±9.8
LV ejection fraction, %	60±5	65±6	5±5
Values Indexed to body surface area			
Indexed LV mass, g/m^2	44±9	45±10	0.6±6.3
Indexed LV end-diastolic volume, mL/m^2	75±12	70±11	-5±6
Indexed LV end-systolic volume, mL/m^2	30±7	25±7	-5±4
Indexed LV stroke volume, mL/m^2	45±7	45±7	0.1±5.7
**CMR RV Measurements**			
Absolute values			
RV end-diastolic volume, mL	143±35	125±31	-19±16
RV end-systolic volume, mL	67±22	49±19	-18±14
RV stroke volume, mL	77±16	76±17	-1±11
RV ejection fraction, %	54±7	61±8	7±7
Values Indexed to body surface area			
Indexed RV end-diastolic volume, mL/m^2	84±15	73±14	-11±9
Indexed RV end-systolic volume, mL/m^2	39±11	29±10	-10±8
Indexed RV stroke volume, mL/m^2	45±7	44±8	-0.7±6.2
**CMR LA and RA Measurements**			
Absolute values			
LA area (4 chamber), cm^2	22±4	19±3	-2.8±2.0
RA area (4 chamber), cm^2	23±4	18±4	-3.0±2.0
Values Indexed to body surface area			
Indexed LA area (4 chamber), cm^2/m^2	13±2	11±2	-1.7±1.2
Indexed RA area (4 chamber), cm^2/m^2	12±2	10±2	-1.7±1.3
**CMR Aortic Root Measurements**			
Absolute values			
Annular diameter, mm	21±2	20±2	-1.0±0.1
Sinus diameter, mm	29±4	29±4	0.4±2.0
Sinotubular junction, mm	23±3	24±4	0.7±1.8
Values Indexed to body surface area			
Indexed annular diameter, mm/m^2	12±1	12±1	-0.3±0.9
Indexed sinus diameter, mm/m^2	17±2	17±2	0.3±1.2
Indexed sinotubular junction, mm/m^2	14±2	13±2	-0.4±1.0
