# Supplementary material for: Cardiovascular magnetic resonance reference ranges for the heart and aorta in Chinese at 3T
Source: J Cardiovasc Magn Reson. 2016 Apr 12;18:21. doi: 10.1186/s12968-016-0236-3 (PMC4830061; doi:10.1186/s12968-016-0236-3)

Correlation Between Phase Contrast and Volumetric Stroke Volumes

We have assessed left ventricular stroke volumes using phase contrast imaging in all individuals in the study. There was excellent correlation between phase contrast and volumetric stroke volumes (r=0.84; P<0.0001), without a significant sex-related difference (**Males**: r=0.78; P<0.0001 and **Females**: r=0.82; P<0.0001).

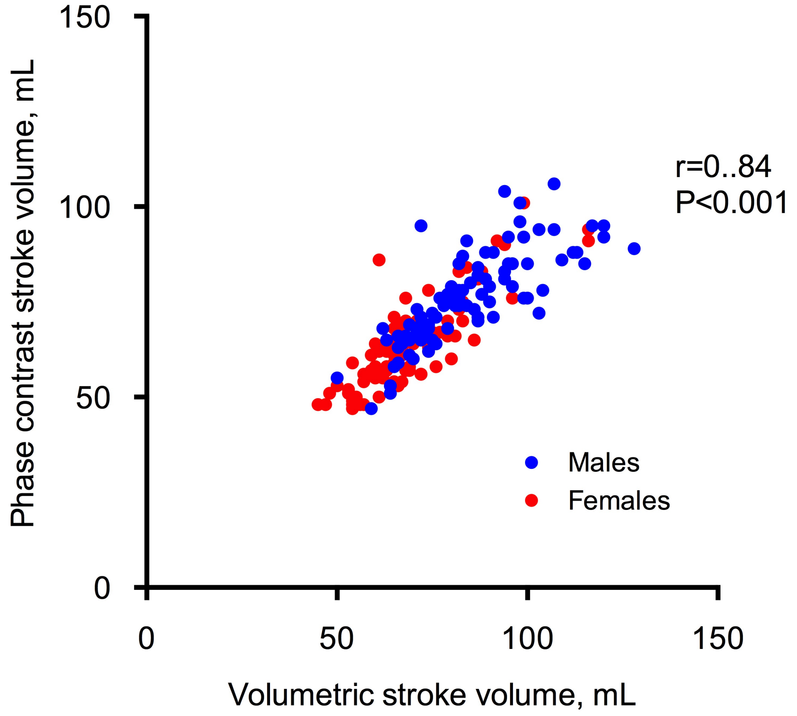

Supplement: Additional file 2: — Correlation between volumetric and phase contrast stroke volumes. (PDF 419 kb) [file 12968_2016_236_MOESM2_ESM.pdf]
